# Computer-aided drug design for the double-membrane vesicle pore complex inhibitors against SARS-CoV-2

**DOI:** 10.3389/fmicb.2025.1562187

**Published:** 2025-03-28

**Authors:** Wang Han, Ruiyuan Zhou, Ruolan Wang, Yanjun Dong, Zeeshan Muhammad, Bin Wang, Jianjun Geng, Haidong Wang, Wei Hou

**Affiliations:** College of Veterinary Medicine, Shanxi Agricultural University, Jinzhong, Shanxi, China

**Keywords:** SARS-CoV-2, double-membrane vesicle pore complex, computer-aided drug design, molecular docking, antiviral agents

## Abstract

Severe acute respiratory syndrome coronavirus 2 (SARS-CoV-2), the etiological agent of the ongoing global pandemic, has constituted the worst global health disaster in recent years. However, no antiviral drugs have proved clinically efficacious to combat SARS-CoV-2 infection. The SARS-CoV-2 double-membrane vesicle (DMV) pore complex, particularly for its positively charged residues R1613, R1614, R303, R305, and R306, which are highly conserved across β-coronaviruses and play a critical role in mediating RNA transport and virus replication, has been validated as an effective drug target. Here, we employed computer-aided drug design (CADD) techniques for the first time to screen the antiviral compounds against SARS-CoV-2 by targeting the crystal structure of the SARS-CoV-2 DMV nsp3-4 pore complex. A total of 486,387 drug compounds were subjected to virtual screening, such as toxicity prediction, ADMET prediction, molecular docking, and target analysis. The six compounds (three for each binding site) were selected based on their lowest binding energies. Notably, Compound 391 demonstrated the strongest binding affinity to the critical positively charged residues R1613 and R1614 at binding site 1, meanwhile, Compound 5,157 exhibited the most stable interactions with the essential positively charged residues R303, R305, and R306 at binding site 2. These results demonstrate that Compound 391 and Compound 5,157 exhibit greater potential antiviral effects, which provide a theoretical basis for further confirmation against SARS-CoV-2 *in vitro* and *in vivo* studies.

## 1 Introduction

Severe acute respiratory syndrome coronavirus 2 (SARS-CoV-2), the etiological agent of the ongoing global pandemic, has constituted the worst global health disaster in recent years, with over six million deaths and more than 768 million confirmed cases globally (Funk et al., 2024). Notably, broad vaccination against SARS-CoV-2 provides immunity for a limited time, and the emergence of new SARS-CoV-2 variants with genetic mutations has raised concerns about the effectiveness of COVID-19 antiviral vaccines, as the variants can partially or completely evade immune responses obtained through vaccination or infection (Edwards et al., 2022; Einav et al., 2021; John et al., 2021). Besides vaccines, developing antiviral drugs that target SARS-CoV-2 is crucial for averting future COVID epidemics. Currently, the approved drugs that inhibit SARS-CoV-2 infection are widely used, such as the RNA polymerase inhibitors Remdesivir, Molnupiravir, and Paxlovid (Bogacheva et al., 2024; Danyu and Thomas, 2023; Robert et al., 2020). However, there are still no effective antiviral drugs that could treat patients or prevent virus transmission, and thus, the search for effective treatments continues.

SARS-CoV-2, as well as other coronaviruses, utilize host membranes to generate viral replication organelles (ROs), inducing either invaginated spherules or double-membrane vesicles (DMVs) to support viral RNA synthesis (Keisuke et al., 2021; Simona et al., 2022; Wolff et al., 2020). In the latter context, the pore complex of DMV is a particularly promising drug target (Huang et al., 2024). SARS-CoV-2 has a relatively large RNA genome, with over two-thirds of it (approximately 20 kb) encoding 16 non-structural proteins (nsp) (Rohitash et al., 2021; Sareh et al., 2023; Tamayo-Ordóñez et al., 2023). Among these, nsp3 and nsp4 have been determined to be the essential minimal viral elements required to form the DMV pore complex (Angelini et al., 2013; Mingming et al., 2022; Oudshoorn et al., 2017; Sakai et al., 2017). Through cryo-ET and subtomogram averaging, nsp3 and nsp4 were identified to exhibit a 6-fold symmetry and form the three constriction sites of the DMV pore. The nsp3-Y1 domain with R1613/R1614 forms the top constriction site, the nsp4 transmembrane domain (TMD) with R303, R305, and R306 forms the central pore site, and the nsp4 carboxy-terminal domain (CTD) with K450 and K452 forms the bottom pore site (Huang et al., 2024). These positively charged residues are highly conserved across β-coronaviruses and play a critical role in mediating RNA transport and virus replication, thus targeting these residues to develop novel antiviral drugs has attracted much attention (Marcus et al., 2019).

Computer-aided drug design (CADD) techniques such as pharmacophore modeling, virtual screening, molecular docking, and dynamic simulation approaches are frequently employed to develop potential drugs to prevent and control virus infection (Farhani et al., 2024; Villanueva, 2022; Zhili et al., 2023). For example, by targeting the crystal structure of the mature Chikungunya virus (CHIKV) envelope protein, CADD was able to identify the natural antiviral agents and ultimately reveal a suitable pocket for designing virus entrance inhibitors (Battini et al., 2021). In addition, to create antiviral treatments against the porcine epidemic diarrhea virus (PEDV), CADD is also utilized to create chemicals that can target the crystallized structure of the 3CL protease (Pathak et al., 2023). Moreover, to discover the potent drugs, CADD focuses on two main approaches: structure-guided and ligand-guided, and when the crystal structure of the protein target is available, structure-guided techniques are preferable (Mehra et al., 2015).

In this study, we employed CADD technology for the first time to screen antiviral compounds against SARS-CoV-2 by targeting the crystal structure of the SARS-CoV-2 DMV nsp3-4 pore complex. After screening, the results revealed that Compound 391 and Compound 5,157 have provided a potential antiviral effect, providing a theoretical basis for further confirmation against SARS-CoV-2 *in vitro* and *in vivo* studies.

## 2 Materials and methods

**FIGURE 1:**
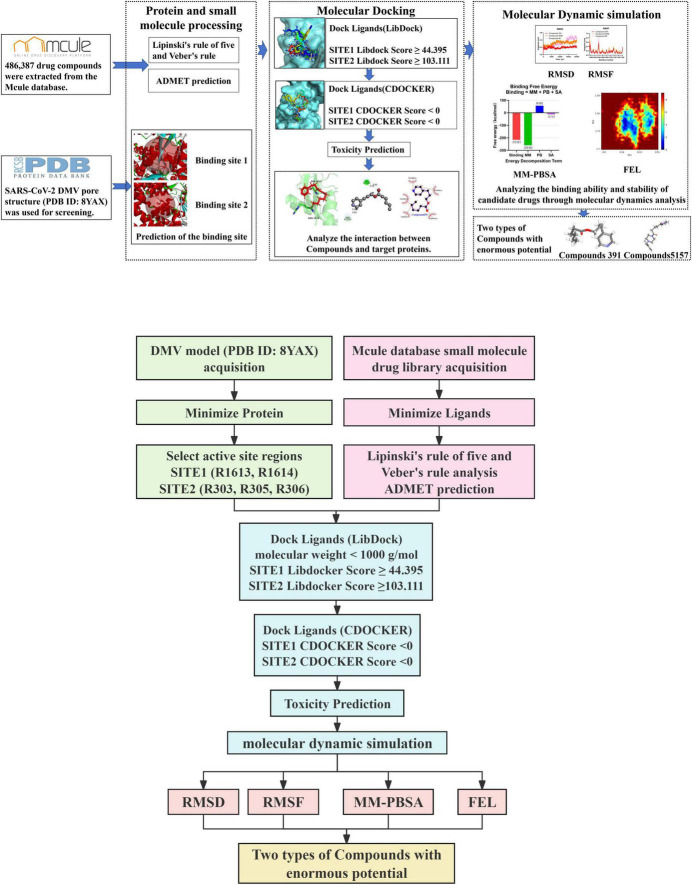
Computational workflow for identifying potential inhibitors targeting the severe acute respiratory syndrome coronavirus 2 (SARS-CoV-2) double-membrane vesicle (DMV) pore complex.

To design potential inhibitors targeting the SARS-CoV-2 double-membrane vesicle (DMV) pore complex using CADD technology, procedures listed in Figure 1 were implemented. In this section, these procedures are briefly mentioned below.

### 2.1 Protein target preparation

To select the available potent inhibitors to the pore structure of SARS-CoV-2 DMV, the crystal structure (PDB ID: 8YAX) of the SARS-CoV-2 DMV nsp3-4 pore complex (full pore) was obtained for virtual screening (Huang et al., 2024). The structure was prepared for drug screening by eliminating its surrounding heteroatoms, side chains, and water molecules, then adding hydrogen and reducing energy using Discovery Studio 4.5 software. The CHARMM force field was applied for energy minimization (Sakae and Okamoto, 2013). After minimizing energy, the binding sites were analyzed through the Define Site tool of Discovery Studio 4.5 software according to the residues at the constriction sites of these two layers.

### 2.2 Drug-likeness analysis and ADMET prediction

A total of 486,387 drug compounds were extracted in SDF format from the Mcule database^1^. The compounds were first filtered through the two rules of drug-likeness: the Lipinski rule of five and the Veber rule (Ahmed et al., 2024). Then, the ADMET module of Discovery Studio 4.5 software was used to evaluate the drug compounds to filter them for those with advantageous absorption, distribution, metabolism, excretion, and toxicity properties. Blood-brain barrier (BBB) level 2, cytochrome P450 2D6 level 0, hepatotoxicity level 0, human intestinal absorption level 1, plasma protein binding (PPB) level 0, and aqueous solubility level 3 were used to select the drug compounds.

### 2.3 Molecular docking and toxicity prediction

Molecular docking was first conducted through the LibDock module of Discovery Studio 4.5 software (Ilaghi-Hoseini and Garkani-Nejad, 2022). The binding sites were used as receptors, and the drug compounds selected from ADMET prediction were analyzed as ligands. The selected ligands were prepared using the two processes, “Prepare Ligands” and “Minimize Ligands” (Nan et al., 2021). The Smart Minimizer algorithm and CHARMM force field were applied for ligand energy minimization (Sakae and Okamoto, 2013). The binding sites and the selected ligands were imported to the working circumstance of LibDock for analysis with the default parameters. All ligand poses were ranked according to the LibDock score. Based on the LibDock score, the compound with a LibDock score > 44.395 (at binding site 1) or 103.111 (at binding site 2) and a molecular weight < 1,000 g/mol was selected by the CDOCKER module of Discovery Studio 4.5 software. Based on the interaction energy CDOCKER module provided, different positions of each ligand at the binding sites were analyzed. The compounds exhibiting negative CDOCKER energy values, which indicate stable docking complexes (Wu and Brooks Iii, 2022), were selected for further analysis. The receptor-ligand interaction was examined using Ligplot and Pymol. Furthermore, the TOPKAT module was utilized to evaluate key pharmacological properties, including Ames mutagenicity, rat oral lethal dose half (LD50), rat carcinogenicity under the United States. National Toxicology Program (NTP), development toxicity potential (DTP) properties, and lowest chronic oral observed adverse effect level (LOAEL) for the compounds identified through the CDOCKER module screening.

### 2.4 Molecular dynamics simulation

The molecular dynamics (MD) simulation was carried out using GROMACS 2020.3 (Groningen Machine for Chemical Simulations) to assess the interactions within protein-ligand complexes (Fu et al., 2022; Jeffrey et al., 2016). Initially, the architecture of the protein-ligand complex was constructed with the CHARMM36 force field. Next, the complex was solvated in a cubic box with TIP3P water, maintaining a minimum distance of 12 Å from the box edge (Emiliani et al., 2022). Sodium (Na^+^) and chloride (Cl^–^) ions were added to neutralize the protein-ligand complex via the genion tool. Subsequently, to prevent steric clashes and incorrect geometries, the solvated and electroneutral system was relaxed through energy minimization through 50,000 steps of the steepest descent algorithm (Eslam et al., 2022). Then, the system equilibration was performed with 100 ps of NVT [substance (N), volume (V), and temperature (T)] and 100 ps of NPT [substance (N), pressure (P), and temperature (T)] without restraints. Afterward, the MD simulation was performed for 20 ns. The RMSD (root mean square deviation), RMSF (root mean square fluctuation), and radius of gyration (Rg) were computed to define the interactions of the protein-ligand complex during the simulation (Lamya et al., 2022). To evaluate binding affinities and intermolecular interactions, binding free energies were calculated using the MM-PBSA method, which combines molecular mechanics (MM), the Poisson-Boltzmann (PB) model, and surface area (SA) calculations. Representative trajectories from the last 15–20 ns of MD simulations (GROMACS) were used, with five snapshots extracted at 1 ns interval from the final 5 ns for analysis (Pathak et al., 2023). Calculations were performed using the gmx mmpbsa.bsh script^2^, with statistical analysis of energy components (MM, PB, SA) to identify key binding contributors and elucidate recognition mechanisms.

## 3 Results and discussion

### 3.1 Prediction of the binding site of SARS-CoV-2 DMV nsp3-4 pore complex

**FIGURE 2:**
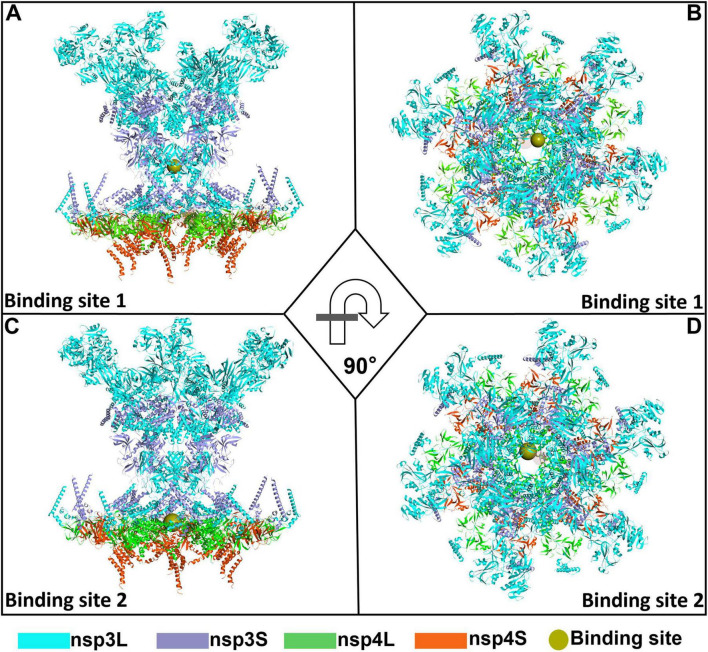
An overall representation map of the predicted binding sites 1 and 2 in the severe acute respiratory syndrome coronavirus 2 (SARS-CoV-2) double-membrane vesicle (DMV) nsp3-4 pore complex. Two orthorhombic views showing the binding site 1 **(A,B)** and binding site 2 **(C,D)** on SARS-CoV-2 DMV nsp3-4 pore complex.

To virtually screen potent drugs against SARS-CoV-2, the crystal structure of the SARS-CoV-2 DMV nsp3-4 pore complex (full pore) was employed in this study. As depicted in Figure 1, in this structure, nsp3 and nsp4 are denoted, respectively as nsp3L (long form), nsp3S (short form), nsp4L (full-length), and nsp4S (short form with the CTD not visible). The structure consists of two layers from top to bottom, generated by nsp3-Y1 and nsp4 TMHs, respectively. R1613/1614 of the nsp3-Y1 domain hexamer forms the first constriction site, while R303, R305, and R306 at the nsp4 TM2–TM3 junction form the second pore (Huang et al., 2024). The positively charged residues R1613, R1614, R303, R305, and R306 were selected as the target positions for predicting ligand binding sites. Using the Define Site from Current Selection module in Discovery Studio 4.5 software, two grid boxes were predicted. One grid box has dimensions of X = 157.6 Å, Y = 146.6 Å, and Z = 113.4 Å (binding site 1), as shown in Figures 2A, B. The other has dimensions of X = 162.8 Å, Y = 108.8 Å, and Z = 164.5 Å (binding site 2), as illustrated in Figures 2C, D.

### 3.2 Screening of the initial molecules

**FIGURE 3:**
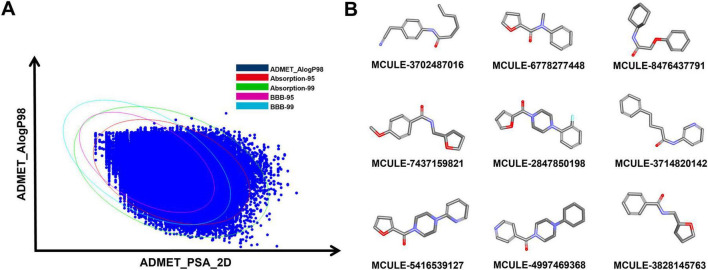
Screening of initial molecules. **(A)** Graph of the screening performance of the ADMET. **(B)** 2D representation of the top 9 molecules through initial screening.

To screen the molecules with drug-likeness, 486,387 compounds from the Mcule database were predicted using Lipinski’s rule of five and Veber’s rule. According to Lipinski’s rule, orally active medication is defined as having several hydrogen bonds acceptor = 10, HBD = 5, MW < 500 Da, and LogP (the logarithm of the octanol-water partition coefficient) = 5 (Yadav et al., 2021). Veber’s rule identifies substances with high oral bioavailability based on two parameters: less than 10 rotatable bonds (ROTB) and a polar surface area (PSA) of less than 140 Å (Yadav et al., 2021). After the screening procedure, a total of 454,810 molecules were obtained. Subsequently, these molecules were filtered based on their ADMET characteristics. The specific criteria were as follows: (1) an aqueous solubility level that was greater than or equal to three; (2) a blood-brain barrier (BBB) level not exceeding two; (3) a cytochrome P450 2D6 level precisely equal to zero; (4) absence of hepatotoxicity, indicated by a level of zero; (5) a human-intestinal absorption level of 1 or lower; (6) a plasma protein binding (PPB) level of zero (Keisuke et al., 2021; Nan et al., 2021). Finally, 8,973 initial molecules were procured (Figure 3A). The top nine representative molecules among them are shown in Figure 3B and ranked in Supplementary Table 1.

### 3.3 Molecular docking-based molecular virtual screening

**FIGURE 4:**
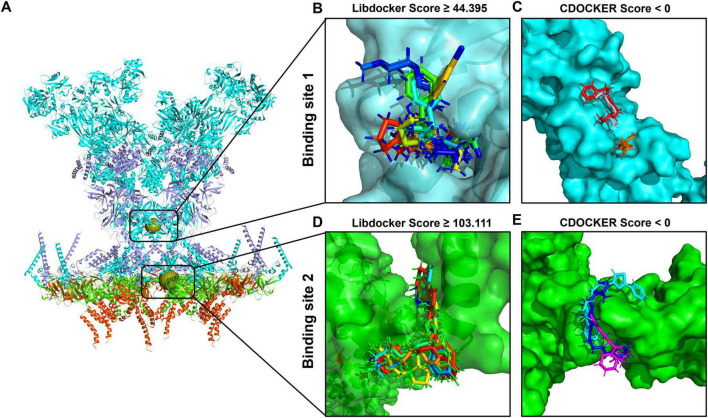
Molecular docking-based molecular virtual screening. **(A)** The structure of the severe acute respiratory syndrome coronavirus 2 (SARS-CoV-2) double-membrane vesicle (DMV) nsp3-4 pore complex, with two binding sites indicated as spherical positions. The surfaces of compounds with the best poses sorted by LibDock and CDOCKER scores are shown in different colors at binding site 1 **(B,C)** and binding site 2 **(D,E)**, respectively.

In drug development, molecular docking is a well-established *in silico*, structure-based approach (Pinzi and Rastelli, 2019). By employing a number of biological, mathematical, and computer-based models, it is able to estimate the affinity of small molecule compounds to target proteins and sift residual interactions between targeting ligands and the receptor active region (Ferreira et al., 2015; Gupta et al., 2018). To filter out the molecules that can bind at the binding site of the SARS-CoV-2 DMV nsp3-4 pore complex, molecular docking was performed through the LibDock and CDOCKER modules. During molecular docking, the two binding sites were used as the receptors and the 8,973 initial molecules as the ligands. A total of 66 and 243,929 docking poses at the binding sites 1 and 2, respectively, were obtained. After screening, 10 docked poses (nine compounds) at the binding site 1 were chosen according to a LibDock score exceeding 44.395 and a molecular weight less than 1,000 g/mol, as shown in Figures 4A, B. Similarly, at the binding site 2, 10 docked poses (10 compounds) were selected with a LibDock score greater than 103.111 and a molecular weight under 1,000 g/mol, as shown in Figures 4A, D. All the compounds chosen based on the Libdock score for each of the two binding sites are listed in Supplementary Table 2.

Subsequently, the selected compounds were further evaluated via the CDOCKER module. Based on the CDOCKER energy, six compounds were selected for their negative interaction energies, as values above 0 kcal/mol are rarely considered reliable indicators of docking stability (Wu and Brooks Iii, 2022). These included three compounds binding to site 1 (Compound 391, Compound 5,568, and Compound 96) and three compounds binding to site 2 (Compound 5,157, Compound 7,052, and Compound 4,149), with detailed results present in Supplementary Table 3. At binding site 1, the lowest CDOCKER interaction energy values are as follows: –24.55 kcal/mol for Compound 391 (colored in red), –20.13 kcal/mol for Compound 5,568 (colored in orange), and –19.29 kcal/mol for Compound 96 (colored in pink), as shown in Figure 4C. In the case of binding site 2, the lowest CDOCKER energy values are –14.32 kcal/mol for Compound 5,157 (colored in blue), –11.43 kcal/mol for Compound 7,052 (colored in magenta), and –3.04 kcal/mol for Compound 4,149 (colored in cyan), as shown in Figure 4E.

To evaluate the safety profiles of the six compounds, the TOPKAT module in Discovery Studio 4.5 software was utilized to predict various toxicity indicators, including rodent carcinogenicity, Ames mutagenicity, DTP, rat oral LD50, and rat chronic (LOAEL) level (Supplementary Table 4). Computational modeling results indicate that all compounds exhibit no carcinogenic effects in either male or female rats, and demonstrate minimal or no mutagenic and developmental toxicity potential. Acute toxicity assessments reveal their rat oral LD50 values ranging from 0.0350 to 3.5301 g/kg body weight, while chronic toxicity evaluations show LOAEL levels of 0.02395 to 0.66195 g/kg body weight for all compounds. Collectively, these findings suggest that the six compounds exhibit favorable safety margins across multiple toxicity domains, supporting their potential as promising candidates for further development.

### 3.4 Analysis of compound interactions at the target binding sites

**FIGURE 5:**
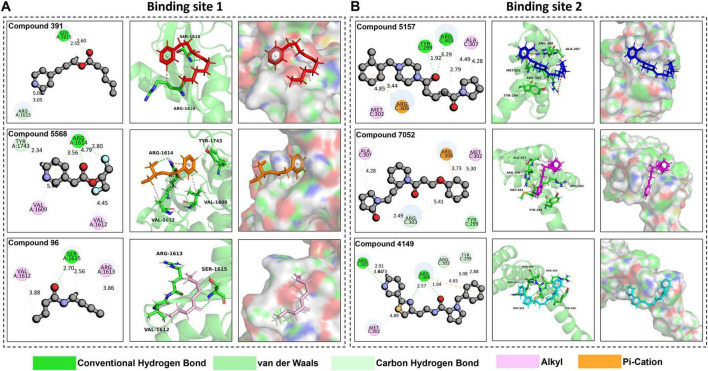
Analysis of compound interaction at target binding sites. **(A)** The interaction interfaces of Compound 391 (colored in red), Compound 5,568 (colored in orange), and Compound 96 (colored in pink) at binding site 1 in 2D and 3D. **(B)** The interaction interfaces of Compound 5,157 (colored in blue), Compound 7,052 (colored in magenta), and Compound 4149 (colored in cyan) at binding site 2 in 2D and 3D.

The compounds interacting with the target binding sites involve various factors, such as binding affinity, the nature of chemical bonds formed (including hydrogen bonds, hydrophobic interactions, electrostatic interactions, etc.), and the spatial orientation of the compounds within the binding sites (Nan et al., 2021; Rojan et al., 2021). These interactions influence the biological activity and functionality of the target binding site and are crucial for drug design and development (Bridges et al., 2023; Fangjiao et al., 2022). The interactions of the compounds at the target binding sites are shown in Figure 5. None of the compounds have unfavorable bonds, which affect the stability of ligand-target binding site complexes due to repulsive forces within the complex. As the significant positively charged residues in the binding site 1 are R1613 and R1614, and in the binding site 2 are R303, R305, and R306 (Huang et al., 2024), the compounds chosen via virtual screening must also possess a potent interaction with these positively charged residues.

As shown in Figure 5A, Compound 391, Compound 5568, and Compound 96 all exhibit favorable interactions with R1613 or R1614 at the binding site 1. Specifically, Compound 391 interacts with R1613 (ARG-1613) through one carbon hydrogen bond and one alkyl. Compound 5,568 interacts with R1614 (ARG-1614) via two conventional hydrogen bonds and one pi-cation. Compound 96 interacts with R1613 (ARG-1613) through an alkyl interaction. Additionally, Compound 391 also interacts with Ser-1615 by forming a conventional hydrogen bond and a carbon hydrogen bond. Compound 5568 has an alkyl interaction with Val-1609 and Val-1612, respectively, and a carbon hydrogen bond with Tyr-1743. Compound 96 interacts with Ser-1615 residues through a conventional hydrogen bond and a carbon hydrogen bond and with Val-1612 residues via an alkyl interaction. Furthermore, Ligplot was also used to analyze the interaction of protein-ligand complexes (Supplementary Figure 1). The findings reveal that these three compounds mainly interact with the target protein by hydrogen and ionic bonds, indicating that these three compounds have excellent performance in tightly binding to active site 1.

Similarly, the three selected compounds, Compound 5,157, Compound 7,052, and Compound 4,149, also interact with the significantly positively charged residues located at binding site 2, as shown in Figure 5B. Compound 5,157 forms an alkyl interaction and a pi-cation interaction with R306 (ARG-306), an alkyl, and two carbon hydrogen bonds with R303 (ARG-303). Additionally, it has an alkyl interaction with MET-302, a carbon-hydrogen bond with Tyr-299, and an alkyl interaction with ALA-307. Compound 7,052 exhibits distinct interaction characteristics. It forms an alkyl interaction and a carbon-hydrogen bond with R303 (ARG-303), a pi-cation interaction with R306 (ARG-306), and a van der Waals interaction with TYR-299. Notably, it also forms an alkyl interaction with both ALA-307 and MET-302, respectively. Compound 4,149 also forms a carbon-hydrogen bond with R303 (ARG-303). When interacting with R305 (ARG-305), Compound 4,149 forms two carbon-hydrogen bonds along with an alkyl interaction. Additionally, it forms two conventional hydrogen bonds and a pi-cation interaction with R306 (ARG-306), a carbon-hydrogen bond with TYR-299, and an alkyl interaction with MET-302. Moreover, protein-ligand interactions were further analyzed using Ligplot (Supplementary Figure 2). The analysis demonstrates that the three compounds primarily interact with the target protein through hydrogen bonds and ionic interactions, suggesting their strong binding affinity to active site 2.

Overall, the interaction of these compounds at target binding sites is stable. Particularly, the interactions between the compounds and the positively charged residues are mainly through hydrogen and ionic bonds, indicating that these compounds exhibit excellent performance in tightly binding to the SARS-CoV-2 DMV nsp3-4 pore complex and have the potential for further study.

### 3.5 Analysis of molecular dynamics simulation

#### 3.5.1 RMSD and RMSF analysis

**FIGURE 6:**
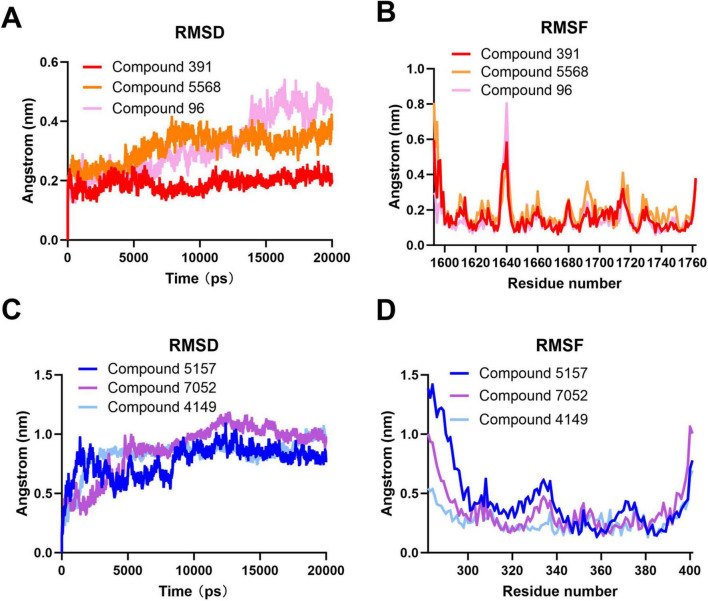
Molecular dynamics simulation of compounds at the binding sites of the severe acute respiratory syndrome coronavirus 2 (SARS-CoV-2) double-membrane vesicle (DMV) nsp3-4 pore complex. **(A)** Root mean square deviation (RMSD) analysis of the compounds at binding site 1. **(B)** Root mean square fluctuation (RMSF) analysis of the compounds at binding site 1. **(C)** RMSD analysis of the compounds at binding site 2. **(D)** RMSF analysis of the compounds at binding site 2.

To determine the dynamic stability of the interaction of the compounds at the target binding sites, a molecular dynamics (MD) simulation of 20 ns was performed on the best docking pose of the compounds with the target protein. As shown in Figure 6A, after running the MD simulation, the complex of Compound 96 exhibits a relatively high RMSD value compared to the complexes of Compound 5,568 and Compound 391 during docking at binding site 1. Among them, the complex of Compound 391 shows the lowest RMSD value. It has been reported that the RMSD of the ligand is fixed within 1 nm, stable below 2 nm, and unstable above 2 nm during molecular docking (Arbaaz et al., 2023; Hou et al., 2023; Sharmin et al., 2021). The RMSD values of three complexes are all below 1 nm, indicating that the binding of all three compounds at binding site 1 is fixed, and the complex of Compound 391 is the most stable. Furthermore, as shown in Figure 6B, the RMSF value, which can evaluate the flexibility of each residue in the docking complex (Lifei et al., 2022; Xiaoxia et al., 2020; Yu et al., 2022), shows that the majority of amino acid residues in the three complexes are less than 0.5 nm. Notably, the few residues displaying significant conformational changes are predominantly located at the periphery of the complex, distal from the compound binding pocket. These results suggest that Compound 391 may serve as a possible lead for the development of antiviral treatments against SARS-CoV-2.

At binding site 2, the complexes of Compound 5,157, Compound 7,052, and Compound 4,149 also performed the MD simulation for 20 ns. As shown in Figure 6C, all three complexes exhibit fewer fluctuations, and the complexes of Compound 7,052 and Compound 4,149 have a higher RMSD value than that of the complex of Compound 5,157, indicating that the binding of Compound 5,157 to the target protein is the most stable. Furthermore, the RMSF values of the three complexes at binding site 2 are comparable to those at binding site 1. As depicted in Figure 6D, most amino acid residues of the three complexes are below 0.5 nm, and only a small number of residues positioned at the complex periphery exhibit significant changes, indicating the rigidity and stability of these three complexes in binding site 2. These results suggest that Compound 5,157 may be another effective inhibitor to restrain the function of the SARS-CoV-2 DMV nsp3-4 pore complex.

#### 3.5.2 Free energy analysis

To further understand the fluctuation of the stable conformation of the protein-ligand during MD simulation, the free energy morphology was mapped, as shown in Supplementary Figure 3. Free energy topography was plotted using the two reaction parameters RMSD (representing protein structural stability during 20 ns of simulation) and the Rg (representing folding state). The energies are colored from higher (red) to lower (blue). For the SARS-CoV-2 DMV nsp3-4 pore complex, energy minima (blue regions) are observed for Compounds 391, 5,568, and 96 at binding site 1, as well as Compounds 5,157, 7,052, and 4,149 at binding site 2. These energy basins indicate that all complexes achieved minimum energy states, representing their most stable conformations.

In addition, to analyze the binding affinity of these compounds at target binding sites, the MM-PBSA binding free energy and residual binding energy calculations were conducted. This method is widely used and recognized for determining the binding free energy of protein-ligand complex data derived from MD simulation results (Genheden and Ryde, 2015; Kumari et al., 2014). The binding free energy calculations were performed using the final 5 ns of the MD simulation trajectories. Positive binding free energy values indicate weak or unfavorable interactions between the ligand and protein, whereas negative values correspond to thermodynamically favorable binding, reflecting stronger molecular interactions (Rahman et al., 2021).

**FIGURE 7:**
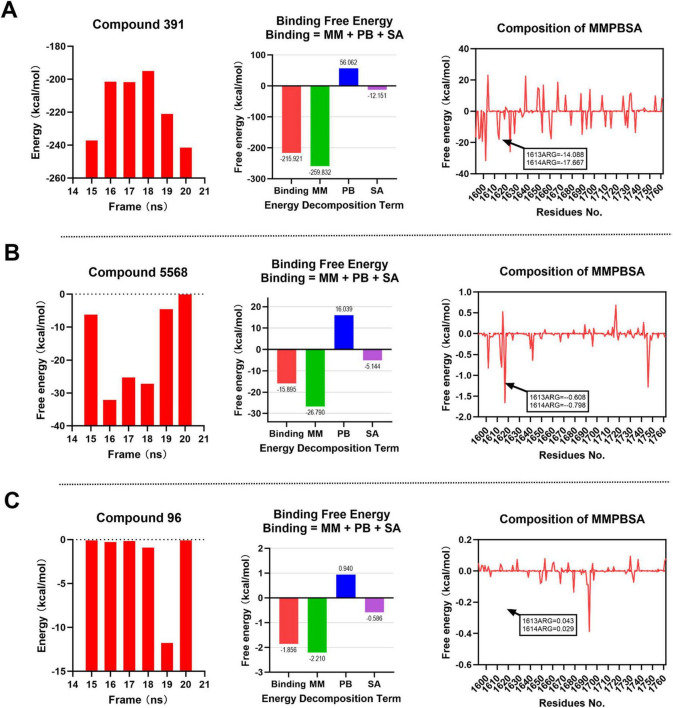
MM-PBSA binding free energy analysis for compounds at binding site 1. Energy component decomposition for Compound 391 complex **(A)**, Compound 5,568 complex **(B)**, and Compound 96 complex **(C)**.

As depicted in Figure 7A, the binding free energy of Compound 391 at binding site 1 remains consistently and markedly below 0 kcal/mol across all time points. The computed average binding free energy for Compound 391 is –215.921 kcal/mol, predominantly driven by the MM component, with supplementary contributions from the SA component. Notably, the binding free energies of Compound 391 with the key amino acid residues R1613 and R1614 are negative, indicating that the interactions between Compound 391 and these two residues are highly stable. As illustrated in Figure 7B, the binding free energies of Compound 5,568 are close to 0 kcal/mol at several time points. It demonstrates an average binding free energy of –15.895 kcal/mol, suggesting a stable binding interaction at binding site 1. However, this stability is not primarily driven by the interactions with the key residues R1613 and R1614, as the binding free energy with these critical amino acids is approximately 0 kcal/mol. In contrast, Compound 96 exhibits the least stable binding at binding site 1, as shown in Figure 7C. Its free energy values at multiple time points are nearly 0 kcal/mol, with an average binding free energy of –1.856 kcal/mol. Additionally, its interaction energy with the key amino acid residues is greater than 0 kcal/mol, indicating a weak and unstable binding. Overall, at binding site 1, Compound 391 demonstrates the most favorable interaction with the key amino acid residues R1613 and R1614, highlighting its superior binding stability and affinity compared to Compounds 5,568 and 96.

**FIGURE 8:**
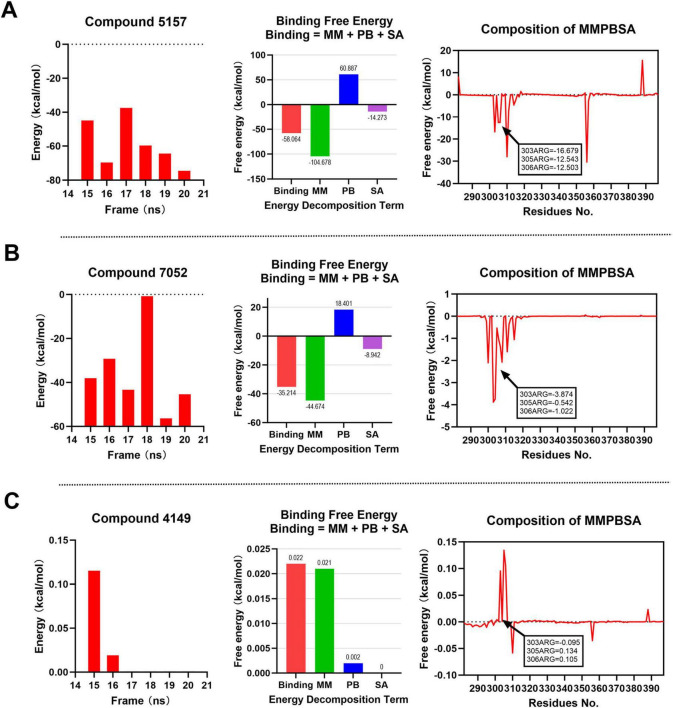
MM-PBSA binding free energy analysis for compounds at binding site 2. Energy component decomposition for Compound 5,157 complex **(A)**, Compound 7,052 complex **(B)**, and Compound 4,149 complex **(C)**.

At binding site 2, as illustrated in Figures 8A, B and Compounds 5,157 and 7,052 demonstrate favorable binding free energies, with values consistently below 0 kcal/mol at each time point. Compound 5,157 exhibits an average binding free energy of –58.064 kcal/mol, while Compound 7,052 shows an average of –35.214 kcal/mol. Notably, both compounds display negative binding free energies with the key amino acid residues R303, R305, and R306, indicating strong and stable interactions with these critical residues, with Compound 5,157 exhibiting particularly remarkable stability. In contrast, as depicted in Figure 8C, Compound 4,149 shows binding free energies that are either greater than or equal to 0 kcal/mol at each time point. Its average binding free energy and interactions with the key amino acids are either positive or close to 0 kcal/mol, suggesting weak and unstable binding at the binding site 2. Therefore, at binding site 2, Compounds 5,157 and 7,052 exhibit favorable binding free energies, with Compound 5,157 demonstrating the most optimal binding effect. This conclusion is consistent with the earlier RMSD and RMSF analyses.

## 4 Limitations and future perspectives

In this study, the CADD technology was employed for the first time to screen the antiviral compounds against SARS-CoV-2 by targeting the crystal structure of the SARS-CoV-2 DMV nsp3-4 pore complex. While our computational approach has provided valuable insights into potential DMV inhibitors targeting SARS-CoV-2, several limitations should be acknowledged to properly contextualize our findings. First, although our ADMET predictions indicate favorable drug-like properties, these computational results require experimental validation to confirm their biological relevance. Second, the accuracy of the predictions is inherently dependent on the quality of the available protein structures and the force fields employed in the simulations. Third, while our study primarily focuses on binding affinity assessments, it may not fully capture the complex dynamics of viral inhibition within cellular environments.

To address these limitations and advance this research, further experimental validation is needed to confirm the findings presented in this study. Specifically, initial *in vitro* testing should include viral replication inhibition assays using SARS-CoV-2-infected host cells, coupled with cytotoxicity evaluations in human cell lines and metabolic stability assessments in liver microsomes. To validate the proposed mechanism of action, surface plasmon resonance (SPR) could be employed for precise binding affinity measurements, complemented by cryo-EM studies to confirm the binding mode and mutational analysis of key binding site residues. For preclinical development, pharmacokinetic profiling in animal models should be conducted, along with efficacy evaluation in SARS-CoV-2-infected animal models and comprehensive toxicology studies to assess safety profiles.

## 5 Conclusion

In this study, the CADD technology was employed for the first time to screen the antiviral compounds against SARS-CoV-2 by targeting the crystal structure of the SARS-CoV-2 DMV nsp3-4 pore complex. A total of 486,387 drug compounds were utilized in the CADD investigations. Following the screening process, Compound 391 and Compound 5,157 were predicted as potential inhibitors that targeted the crucial positively charged residues in the binding sites 1 and 2, respectively. The specific targeting of key residues by Compound 391 and Compound 5,157 suggests a prospective approach for combating SARS-CoV-2. The stability of this interaction signifies that these two compounds may have a lasting effect on inhibiting the virus replication process. Further research could explore the detailed mechanism of action of these compounds and optimize their properties to enhance their inhibitory potency. Additionally, the CADD approach used in this study can be extended to screen other potential compounds and target sites, potentially uncovering additional candidates with anti-SARS-CoV-2 activity.

## Data Availability

The datasets presented in this study can be found in online repositories. The names of the repository/repositories and accession number(s) can be found in the article/Supplementary material.

## Footnotes

1
https://mcule.com/database/

2
https://github.com/Jerkwin/gmxtools/tree/master/gmx_mmpbsa
