# Supplementary material for: Computer-aided drug design for the double-membrane vesicle pore complex inhibitors against SARS-CoV-2
Source: Front Microbiol. 2025 Mar 28;16:1562187. doi: 10.3389/fmicb.2025.1562187 (PMC11985525; doi:10.3389/fmicb.2025.1562187)
Supplement: Supplementary file 1 [file Data_Sheet_1.docx]

Supplementary Material

# Supplementary Tables

**Table S1.** ADMET characteristics of the top 9 compounds selected from the Database

| **Number** | **Molecular ID** | **Solubility^a^** | **BBB^b^** | **Cytochrome P450 2D6 inhibitory activity^c^** | **Hepatotoxicity^d^** | **Absorption^e^** | **PPB^f^** |
| --- | --- | --- | --- | --- | --- | --- | --- |
| 1 | MCULE-3702487016 | 3 | 2 | 0 | 0 | 0 | 0 |
| 2 | MCULE-6778277448 | 3 | 1 | 0 | 0 | 0 | 0 |
| 3 | MCULE-8476437791 | 3 | 1 | 0 | 0 | 0 | 0 |
| 4 | MCULE-7437159821 | 3 | 2 | 0 | 0 | 0 | 0 |
| 5 | MCULE-2847850198 | 3 | 1 | 0 | 0 | 0 | 0 |
| 6 | MCULE-3714820142 | 3 | 2 | 0 | 0 | 0 | 0 |
| 7 | MCULE-5416539127 | 3 | 2 | 0 | 0 | 0 | 0 |
| 8 | MCULE-4997469368 | 3 | 2 | 0 | 0 | 0 | 0 |
| 9 | MCULE-3828145763 | 3 | 2 | 0 | 0 | 0 | 0 |

*^a^ Aqueous-solubility level: 0 (extremely low);1 (very low, but possible); 2 (low); 3 (good); 4 (optimal).*

*^b^ Blood Brain Barrier level: 0 (Very high penetrant); 1 (High); 2 (Medium); 3 (Low); 4 (Undefined).*

*^c^ Cytochrome P450 2D6 level: 0 (Non-inhibitor); 1 (Inhibitor).*

*^d^ Hepatotoxicity: 0 (Nontoxic);1(Toxic).*

*^e^ Human-intestinal absorption level: 0 (good); 1 (moderate); 2 (poor); 3 (very poor).*

*^f^ Plasma Protein Binding: 0 (Absorbent weak);1 (Absorbent strong).*

**Table S2. The compounds selected based on the LibDock Scores at each respective binding site**

| **Site** | **Number** | **Molecular ID** | **LibDock score** |
| --- | --- | --- | --- |
| Site 1 | 1 | MCULE-5675003128 | 50.8242 |
|  | 2 | MCULE-9440619345 | 50.6166 |
|  | 3 | MCULE-2905519788 | 50.2015 |
|  | 4 | MCULE-9972084483 | 49.4047 |
|  | 5 | MCULE-5756937097 | 48.3903 |
|  | 6 | MCULE-1280625603 | 48.0226 |
|  | 7 | MCULE-2738517274 | 47.8889 |
|  | 8 | MCULE-9947899176 | 44.7391 |
|  | 9 | MCULE-7141834161 | 44.395 |
| Site 2 | 1 | MCULE-7433677278 | 114.513 |
|  | 2 | MCULE-7766755230 | 112.174 |
|  | 3 | MCULE-1141822408 | 110.202 |
|  | 4 | MCULE-2961136471 | 107.798 |
|  | 5 | MCULE-9609325001 | 106.11 |
|  | 6 | MCULE-8157563330 | 104.632 |
|  | 7 | MCULE-7753434369 | 104.478 |
|  | 8 | MCULE-7033087753 | 103.374 |
|  | 9 | MCULE-9609325001 | 103.281 |
|  | 10 | MCULE-2052758660 | 103.111 |

**Table S3.** The compounds are presented with their lowest CDOCKER interaction energies at each respective binding site

| **Site** | **Number** | **Compound** | **Molecular ID** | **CDOCKER interaction energy (Kcal/mol)** |
| --- | --- | --- | --- | --- |
| Site 1 | 1 | Compound 391 | MCULE-5675003128 | -24.55 |
|  | 2 | Compound 5568 | MCULE-9440619345 | -20.130 |
|  | 3 | Compound 96 | MCULE-2905519788 | -19.29 |
| Site 2 | 1 | Compound 5157 | MCULE-7433677278 | -14.32 |
|  | 2 | Compound 7052 | MCULE-7766755230 | -11.43 |
|  | 3 | Compound 4149 | MCULE-9609325001 | -3.04 |

**Table S4.** Toxicities of Compounds

| **Name** | **TOPKAT Rat Female NTP Prediction^a^** | **TOPKAT Rat Male NTP Prediction^b^** | **TOPKAT Ames Prediction^c^** | **TOPKAT DTP Prediction^d^** | **TOPKAT Rat Oral LD50^e^** | **TOPKAT Chronic LOAEL^f^** |
| --- | --- | --- | --- | --- | --- | --- |
| Compound391 | 0.213861 | 0.286494 | 0.00884086 | 0.451365 | 2.5669 | 0.66195 |
| Compound5568 | 0.286494 | 0.560182 | 0.528243 | 0.414197 | 0.0350 | 0.02395 |
| Compound96 | 0.518339 | 0.295554 | 0.42969 | 0.445927 | 3.5301 | 0.18706 |
| Compound5157 | 0.560182 | 0.448604 | 0.396722 | 0.585447 | 1.7020 | 0.16805 |
| Compound7052 | 0.286543 | 0.148177 | 0.185053 | 0.555955 | 1.6348 | 0.02584 |
| Compound4149 | 0.295554 | 0.319428 | 0.621101 | 0.331295 | 0.8402 | 0.05924 |

*^a^ NTP <0.3 (noncarcinogen); >0.8 (carcinogen).*

*^b^ NTP <0.3 (noncarcinogen); >0.8 (carcinogen).*

*^c^ Ames <0.3 (nonmutagen); >0.8 (mutagen).*

*^d^ DTP <0.3 (nontoxic); >0.8 (toxic).*

*^e^ Rat Oral LD50 is expressed in g/kg body weight.*

*^f^ Chronic LOAEL is expressed in g/kg body weight.*

# Supplementary Figures


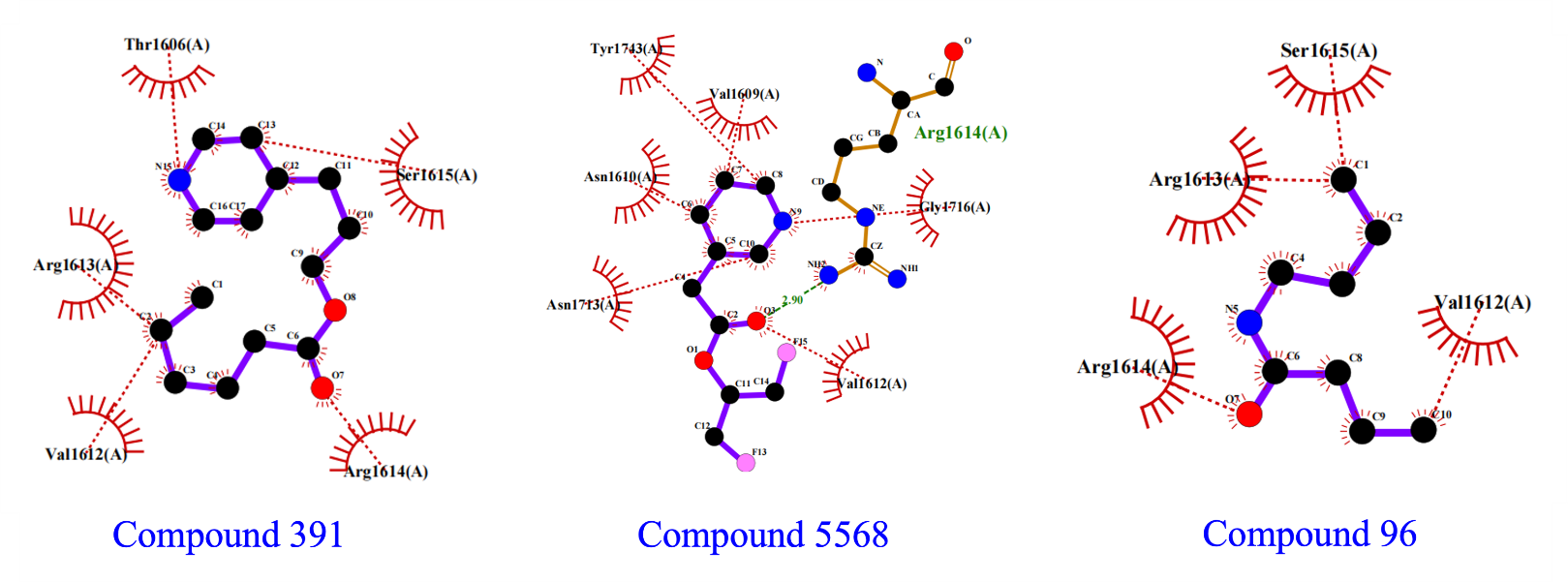


**Figure S1.** **Two-dimensional interaction diagrams of Compound 391, Compound 5568, and Compound 96 at binding site 1 generated by Ligplot.** The dotted green and red lines represent hydrogen and hydrophobic bonds, respectively.


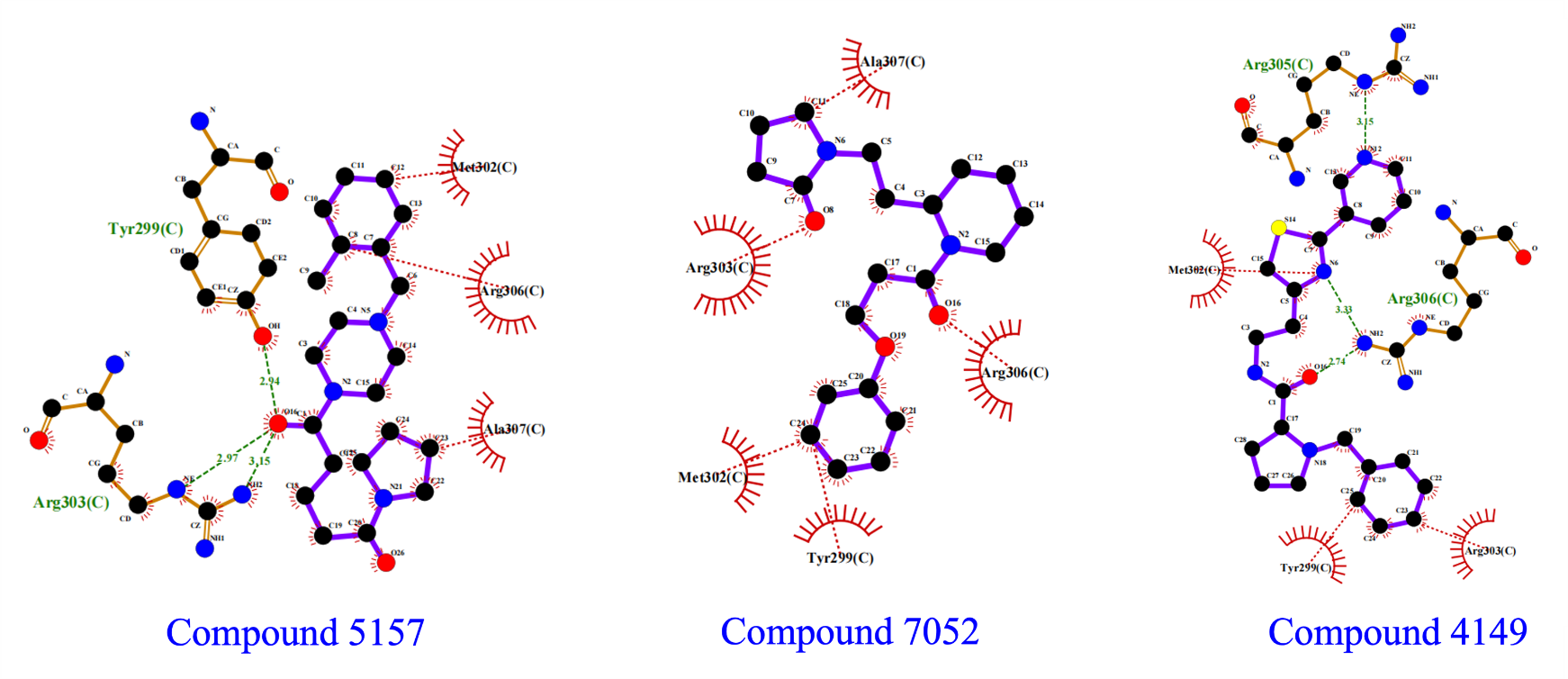


**Figure S2.** **Two-dimensional interaction diagrams of Compound 5157, Compound 7052, and Compound 4149 at binding site 2 generated by Ligplot.** The dotted green and red lines represent hydrogen and hydrophobic bonds, respectively.


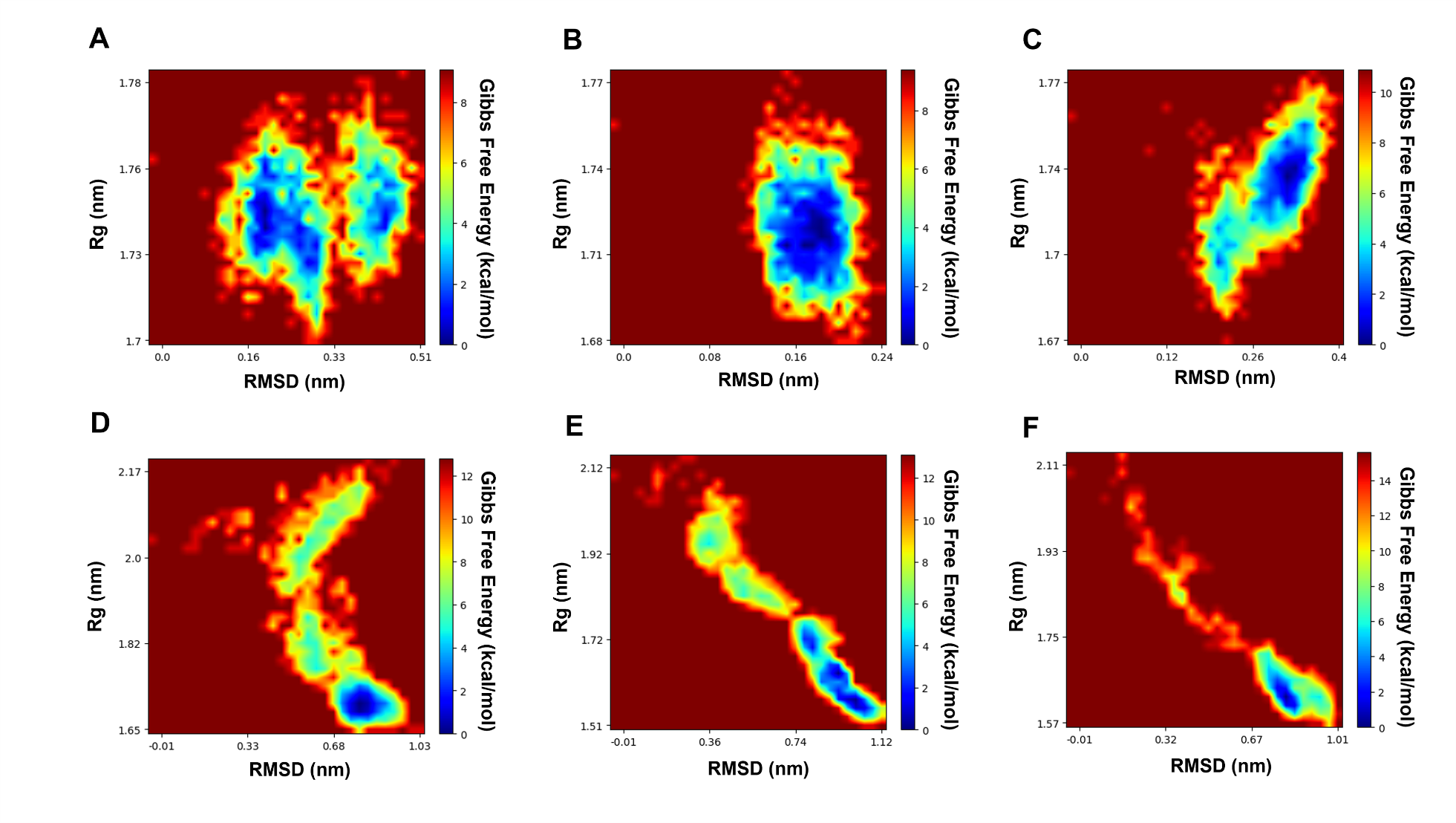


**Figure S3.** **The color-coded illustration of the Gibbs free energy landscape (FEL) was plotted using Rg and RMSD.** (A)-(C) represent the FEL for binding site 1 complexes with Compound 391, Compound 5568, and Compound 96, respectively. (D)-(F) represent the FEL for binding site 2 complexes with Compound 5157, Compound 7052, and Compound 4149, respectively.
